# Microbial profile of symptomatic pericoronitis lesions: a cross-sectional study

**DOI:** 10.1590/1678-7757-2019-0266

**Published:** 2019-11-15

**Authors:** Marcus Heleno Borges Ribeiro, Paulo Cesar Ribeiro, Belén Retamal-Valdes, Magda Feres, Antonio Canabarro

**Affiliations:** 1 Universidade Iguaçu, Curso de Odontologia, Departamento de Cirurgia Oral, Nova Iguaçu, Rio de Janeiro, Brasil.; 2 Universidade Guarulhos, Divisão de Pesquisa Odontológica, Departamento de Periodontia, Guarulhos, São Paulo, Brasil.; 3 Universidade Veiga de Almeida, Programa de Pós-Graduação em Odontologia, Rio de Janeiro, Rio de Janeiro, Brasil.; 4 Universidade Estado do Rio de Janeiro, Departamento de Procedimentos Clínicos Integrados, Rio de Janeiro, Rio de Janeiro, Brasil.

**Keywords:** Pericoronitis, Periodontitis, Microbiota

## Abstract

**Objective:**

The microbial composition of pericoronitis (Pc) is still controversial; it is not yet clear if the microbial profile of these lesions is similar to the profile observed in periodontitis (Pd). Therefore, the aim of the present study was to describe the microbial profile of Pc lesions and compare it directly with that of subjects with Pd.

**Methodology:**

Subjects with Pc and Pd were selected, and subgingival biofilm samples were collected from (i) third molars with symptomatic Pc (Pc-T), (ii) contralateral third molars without Pc (Pc-C) and (iii) teeth with a probing depth >3 mm from subjects with Pd. Counts and proportions of 40 bacterial species were evaluated using a checkerboard DNA-DNA hybridization technique.

**Results:**

Twenty-six patients with Pc and 18 with Pd were included in the study. In general, higher levels of microorganisms were observed in Pd. Only *Actinomyces oris* and *Eubacterium nodatum* were present in higher mean counts in the Pc-T group in comparison with the Pc-C and Pd-C groups (p<0.05). The microbiota associated with Pc-T was similar to that found in Pc-C. Sites with Pc lesions had lower proportions of red complex in comparison with the Pd sites.

**Conclusion:**

The microbiota of Pc is very diverse, but these lesions harbour lower levels of periodontal pathogens than Pd.

## Introduction

Pericoronitis (Pc) is an infectious condition involving the soft tissue around the crown of a partially erupted tooth.^1^ Thus, a high prevalence of Pc during the eruption of primary and permanent dentition could be expected. However, this condition rarely occurs in primary dentition; it is mainly associated with the eruption of the mandibular third molars^2^ and is more commonly reported in females.^3^ Although Pc may affect the patient's quality of life because it is often followed by discomfort, pain, bleeding, halitosis or even trismus, this condition is often neglected in daily clinical practice.^1^

Third-molar eruption normally occurs in people between 18 and 25 years old, but problems with the eruption process are frequently observed.^2^ A study that evaluated 245 cases of Pc found 35% of these cases occurred in patients between 20 and 29 years old. The occlusal surface of the affected tooth is often covered by gingival tissue, which favors the accumulation of food and biofilm, promoting the development of an infectious process.^2^ Vertically impacted molars are more likely to present Pc.^4^ Severe cases have an associated risk of systemic dissemination of the infection.^5^

Very few studies to date have analyzed the microbial composition associated with Pc.^2^^,^^6^^–^^11^ Overall, studies have shown that periodontal pathogens are common in third-molar periodontal sites in subjects without periodontal diseases.^9^^–^^11^ Previous studies have demonstrated the presence of gram-negative anaerobes and mobile forms of spirochetes in periodontal sites with Pc,^6^^,^^7^ and concluded that the composition of the biofilm associated with Pc seems to be similar to that found in periodontitis (Pd).^8^^–^^10^ However, these studies evaluated only a few biofilm samples and microbial species^6^^,^^7^ or were not Pc patients and Pd patients comparative studies,^8^^–^^11^ which could preclude a complete understanding on the microbial profile of Pc. Therefore, this study aims to describe the microbial profile of Pc lesions and compare it directly with the microbial profile of subjects with Pd.

## Methodology

### Study design and settings

This is a bicentric cross-sectional study with a control-to-case ratio of 0.7. This study was conducted at Veiga de Almeida University (Universidade Veiga de Almeida – UVA, Rio de Janeiro, RJ, Brazil) and Iguaçu University (Universidade Iguaçu - UNIG, Nova Iguaçu, RJ, Brazil) from January to March 2015. The study protocol was previously approved by the Human Research Ethics Committee of Veiga de Almeida University, Rio de Janeiro, RJ, Brazil (protocol number 962399).

### Participants

Systemically healthy volunteers diagnosed with untreated Pc or Pd were selected from the population that searched for dental treatment. Subjects who fulfilled the inclusion criteria were invited to participate in the study. All eligible subjects were thoroughly informed of the nature, potential risks, and benefits of their participation in the study and then signed an Informed Consent Form. Detailed medical and dental histories were obtained, and a full-mouth periodontal examination was performed.

The inclusion criteria for the study groups were: (I) Pericoronitis test (Pc-T): at least one mandibular third molar with partial eruption and gingival tissue covering the crown of the tooth with one or more of the following symptoms: pain, edema and spontaneous bleeding; (II) Pericoronitis control (Pc-C): at least one contralateral third molar of the same Pc patient with no periodontal pocket depth (PPD<3 mm) and no bleeding on probing. If this tooth was absent, data of contralateral first or second molar were used; and (III) Periodontitis control (Pd-C): adults (>35 years old) with ≥2 teeth with ≥1 detectable buccal or interproximal site with a clinical attachment level (CAL) ≥ 3 mm and a PPD > 3 mm.^12^

The exclusion criteria were: presence of Pd (for Pc groups), traumatic injury of the soft tissues, periodontal treatment in the previous six months, smoking, pregnancy, lactation, use of prostheses, all systemic conditions that could affect the periodontal microbial composition (for example HIV, diabetics, etc.), treatment with nonsteroidal anti-inflammatory drug and antibiotic medications or mouthwashes in the previous six months.

A total of 44 volunteers participated in the study, 26 with Pc (16 females and 10 males, aged between 19 and 29 years old) and 18 with Pd (12 females and 6 males, aged between 35 and 67 years old). The mean ages (±standard deviation) of the Pc and Pd groups were 25.46±2.87 and 48.89±13.02, respectively (p>0.05). Pd also showed a mean PPD of 4.72±1.40 mm.

### Microbiological monitoring - sample collection

Subgingival biofilm samples were collected from two periodontal sites per volunteer, including:

#### Pc groups:

(I) Pc-T – the deepest periodontal site of the mandibular third molar with Pc and (II) Pc-C - one site with a PPD < 3 mm and no bleeding on probing of the mandibular third molar in the contralateral quadrant.

#### Pd group:

(III) the site with the deepest periodontal pocket in the mouth (PPD≥4 mm).

After the clinical examination, all the teeth were dried and isolated using cotton rolls. After supragingival plaque removal, subgingival biofilm samples were collected using sterile paper points (size 45) (Dentsply Sirona, Pirassununga, SP, Brazil) inserted into each site for 30 seconds, as previously described.^13^^,^^14^ The samples were immediately placed into individual tubes containing 150 μl of TE buffer solution (10 mM Tris-HCL; Life Technologies, Carlsbad, CA, USA) and 1 mM of EDTA (Labsynth, Diadema, SP, Brazil; pH 7.6). 100 μl of 0.5 M NaOH (Labsynth) was added to each tube to preserve the bacterial DNA. All the tubes were stored under refrigeration at −20°C until the samples were analyzed using checkerboard DNA-DNA hybridization at the Laboratory of Microbiology, Immunology and Molecular Biology of Universidade de Guarulhos (Guarulhos, SP, Brazil).

### Checkerboard DNA–DNA hybridization

**Figure 1:**
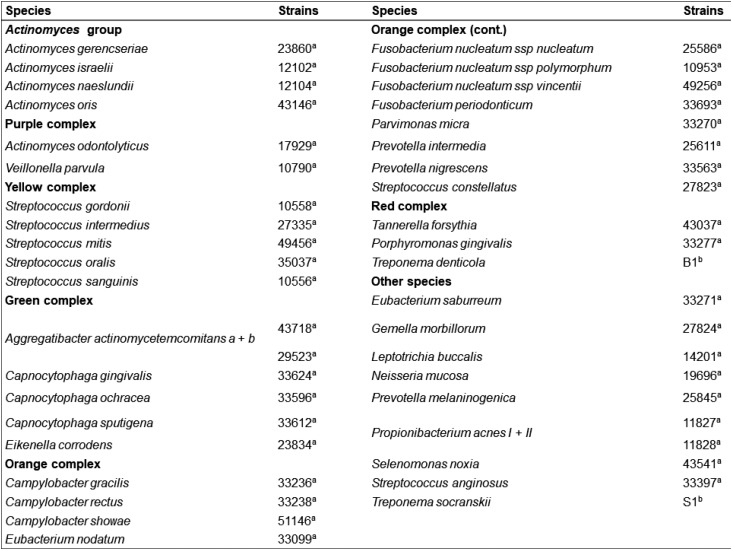
Bacterial strains used for the preparation of DNA probes. Species are grouped according to the microbial complexes^18^

Counts and proportions of 40 bacterial species were determined in each sample, using the checkerboard DNA–DNA hybridization technique.^15^^,^^16^ The samples were boiled for 10 min and neutralized using 0.8 mL of 5 M ammonium acetate. The released DNA was then placed into the extended slots of a Minislot 30 apparatus (Immunetics, Marlborough, MA, USA), concentrated on a 15x15 cm positively charged nylon membrane (Boehringer Mannheim, Indianapolis, IN, USA), and were fixed to the membrane by baking it at 120°C for 20 min. The membrane was then placed in a Miniblotter 45 (Immunetics) with the lanes of DNA at 90° to the lanes of the device. Digoxigenin-labelled whole genomic DNA probes for 40 bacterial species (Figure 1) were hybridized in individual lanes of the Miniblotter. After the hybridization, the membranes were washed at high stringency, and the DNA probes were detected using the antibody to digoxigenin conjugated with alkaline phosphatase. Chemiluminescence detection was then performed. The last two lanes in each run contained standards at concentrations of 10^5^ and 10^6^ cells of each species. Signals were evaluated visually by comparison with the standards at the 10^5^ and 10^6^ bacterial cells for the test species on the same membrane by a calibrated examiner. The sensitivity of this assay was adjusted to allow detection of 10^4^ cells of a given species by adjusting the concentration of each DNA probe. This procedure was carried out to provide the same sensitivity of detection for each species.

### Statistical methods

The mean counts (x10^5^ cells) of the individual bacterial species were averaged within each subject and then across the subjects in the different clinical groups. Similarly, the percentage of the total DNA probe counts was determined initially in each site, then per subject and averaged across the subjects in the three groups. The individual proportions of each species were added to determine the proportions of each microbial complex.^17^ The significance of the differences between the groups was assessed using one-way ANOVA test. In addition, a t-test was used to determine significant differences between the pairs of groups. Adjustments for multiple comparisons were performed when the 40 bacterial species were evaluated simultaneously.^17^ All the analyses of this study were conducted using a statistical program developed by Sigmund Socransky (The Forsyth Institute, Cambridge, MA, USA). The significance level was set at 5%.

## Results

**Figure 2:**
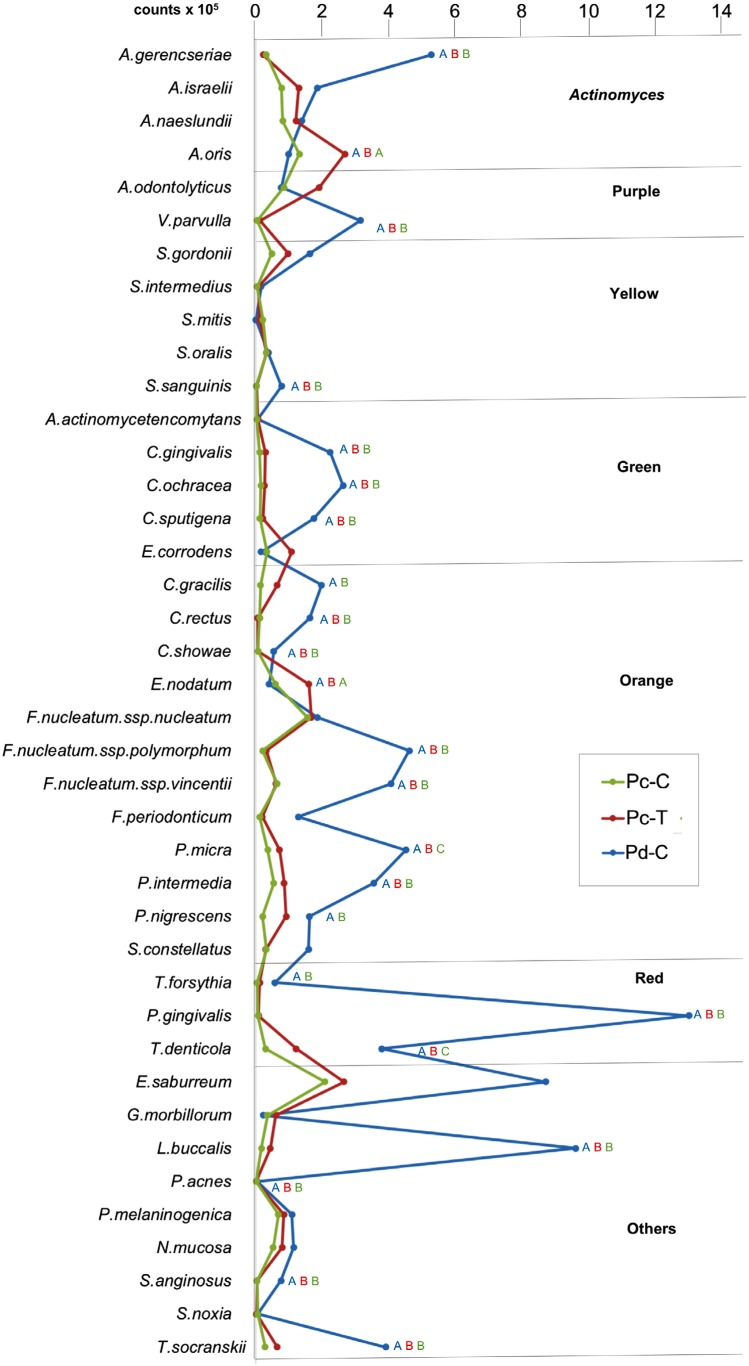
Mean counts (10^5^) of 40 subgingival species in each study group. The species were ordered according to the microbial complexes described by Socransky, et al.^18^ (1988). The significance of differences between groups was assessed using one-way ANOVA test. Different letters indicate significant differences between pairs of groups (t-test, p<0.05). Letters were color coded to indicate the different groups: green for Pc-C, red for Pc-T, and blue for Pd-C. Pc-C: Pericoronitis control group; Pc-T: Pericoronitis test group; Pd-C: Periodontitis control group

Figure 2 shows the mean counts (x10^5^ cells) of the sites colonized by the 40 species evaluated in the subgingival plaque samples from the Pc-C, Pc-T and Pd-C groups. The species present in the highest levels in Pc-T were *Actinomyces oris, Eikenella corrodens, Eubacterium nodatum, Fusobacterium nucleatum spp. nucleatum, Treponema denticola* and *Eubacterium saburreum. A. oris* and *E. nodatum* were present in higher mean counts in the Pc-T group in comparison with the Pc-C and Pd-C groups (p<0.05). The microbiota associated with the Pc-T group was very similar to that found in Pc-C. Most of the bacterial species evaluated in the study were found in higher counts in the Pd-C group, and 20 of them were significantly higher in this group compared with the Pc-T group, including *Actinomyces gerencseriae, A. oris, Veillonella parvula, Streptococcus sanguinis, Capnocytophaga gingivalis, Capnocytophaga ochracea, Capnocytophaga sputigena, Campylobacter rectus*, *Campylobacter showae, E. nodatum, Fusobacterium nucleatum. spp. polymorphum, Fusobacterium nucleatum. spp. vicentii, Parvimonas micra, Prevotella intermedia, Porphyromonas gingivalis, Treponema denticola, Leptotrichia buccalis*, *Propionibacterium acnes, Streptococcus anginosus* and *Treponema socranskii* (p<0.05).

**Figure 3:**
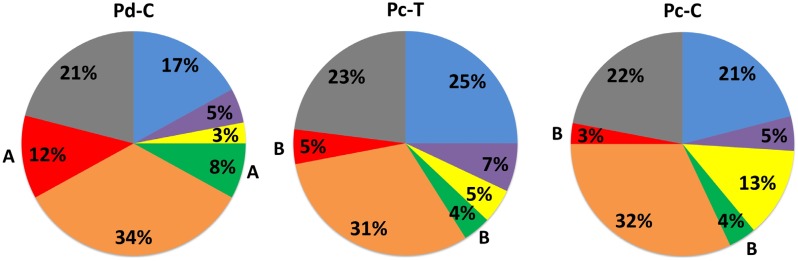
Mean proportions of the microbial complexes in each study group. The colors represent different microbial complexes^18^ and *Actinomyces* species (blue). The significance of differences between groups was sought using the one-way ANOVA. The differences were only found for red and green complexes. Different letters indicate significant differences between pairs of groups (t-test, p<0.05). Pc-C: Pericoronitis control group; Pc-T: Pericoronitis test group; Pd-C: Periodontitis control group

The mean proportions of the microbial complexes in the different groups are described in Figure 3. The red complex pathogens were higher in Pd-C than Pc-T and Pc-C groups (p<0.05). A similar trend was also observed for the green complex (p<0.05). However, a tendency towards a higher proportion of yellow complex species in the PC groups was noticed (p=0.09).

## Discussion

The results of this study showed Pc sites harbored a quite diverse microbiota; nonetheless, with a lower degree of dysbiosis than that observed in Pd lesions. Pc biofilm samples had lower levels and proportions of putative and traditional periodontal pathogens and a tendency towards higher levels of the health-associated yellow complex species than Pd lesions.

The red complex, which harbors the three most traditional periodontal pathogens (*P. gingivalis, T. denticola* and *Tannerella forsythia)*,^18^ was present in higher counts in Pd patients, compared with both the Pc groups. Although previous studies have shown a high number of *T. forsythia* in Pc patients,^11^^,^^19^ this study could not confirm these findings. It is important to highlight that *P. gingivalis*, an anaerobic gram-negative bacteria, and *T. denticola*, an anaerobic gram-negative spirochete, have been considered key periodontal pathogens.^20^ Those are frequently found to co-exist in deep periodontal pockets.^21^ Such interaction between them can contribute to Pd progression.^22^ *T. socranskii*, another anaerobic gram-negative spirochete, is also considered a periodontal pathogen^23^^,^^24^ associated with Pc^25^ and was also found in higher proportions and levels in the Pd group. Interestingly, these 3 microorganisms together (*P. gingivalis*, *T. denticola*, and *T. socranskii*) were correlated with abnormal periodontal clinical parameters and have been associated with periodontal tissue loss.^23^

In fact, few published studies compared putative pathogens in healthy and Pc sites. Some of these studies have used conventional culture-dependent methods that many times fail to detect strict anaerobe pathogens,^6^^,^^7^ at least one study neglected to include a control group without Pc, hampering the interpretation of the results.^8^ Another study compared healthy and symptomatic Pc sites, and the results supported the hypothesis that the pericoronal region harbors putative periodontal pathogens,^10^ and may provide a favored niche for periodontal pathogens in a healthy oral environment.^9^

Discussing the clinical findings of this study in relation to the microbial profiles observed in the various lesions is important. First, these findings suggest the biofilm associated with Pc apparently does not have a strong potential to trigger irreversible periodontal destruction since it does not harbor high levels of major periodontal pathogens and maintains good levels of host-compatible microorganisms. As Pc is an acute disease, one could hypothesize that time between the development of the lesion and its treatment for the massive growth of key periodontal pathogens was insufficient. Besides that, the prophylactic surgical removal of teeth with Pc is frequently performed in dental practice.^26^ In addition, antibiotic treatment must be considered in patients whose Pc infections were disseminated and have invaded deeper oral spaces.^26^ Most Pc lesions are associated with a subgingival anaerobic niche created by the overgrowth of gingival tissues. Nonetheless, the differences between the microbial load of Pd and Pc shown in this study suggest the antibiotic protocols required to treat these conditions may not be the same. Future studies addressing this topic would help to guide clinical practice.

The main strength of this study is that, to the best of our knowledge, this is the first study to comprehensively assess the microbial composition of Pc lesions and to compare this profile to that found in Pd. One limitation of the study design is the relatively small sample size, due to the difficulty in selecting Pc cases in daily clinical practice. Furthermore, this study shows results for the 40 bacterial species proposed by Socransky, et al.^18^ (1988) as it is well established that the periodontal microbiome comprises more taxa than those included in this group of bacterial species.^27^ Nevertheless, this panel of species has been successfully used as a biological marker for many studies of periodontal disease risk and treatment.^28^^,^^29^ A comprehensive study showed it covers approximately 60% of the bacterial genera present in the oral cavity.^30^

Few studies have identified periodontal bacteria in pericoronitis samples, but it seems that pericoronal sites can harbor several pathogens. A recent study showed some periodontopathic bacteria and herpesviruses occurred concomitantly in pericoronitis samples.^11^ Such herpesviral-bacterial interaction could be an important feature of pericoronitis and should be further studied.

In conclusion, Pc microbiota is diverse, but these lesions harbor lower levels of periodontal pathogens than those of Pd.
